# Pseudomesotheliomatous carcinoma of lung adenocarcinoma diagnosed using transesophageal ultrasound‐guided bronchoscopic aspiration

**DOI:** 10.1111/1759-7714.13916

**Published:** 2021-03-10

**Authors:** Toshiyuki Sumi, Hisashi Nakata, Yuji Mori, Hirofumi Chiba

**Affiliations:** ^1^ Department of Pulmonary Medicine Hakodate Goryoukaku Hospital Hakodate Japan; ^2^ Department of Respiratory Medicine and Allergology Sapporo Medical University School of Medicine Sapporo Japan

**Keywords:** EUS‐B‐FNA, lung cancer, pleural effusion

## Abstract

Endoscopic ultrasound with bronchoscope‐guided fine‐needle aspiration (EUS‐B‐FNA), a minimally invasive procedure, may be useful for the diagnosis of pseudomesotheliomatous (PMC) carcinoma because a sufficient amount of tissue can be obtained for diagnosis. This is the first report of PMC diagnosed using EUS‐B‐FNA. Our findings suggest that EUS‐B‐FNA may reduce the risk of dissemination for PMC.

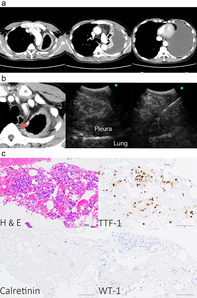

A 66‐year‐old man was admitted with a history of dyspnea. Computed tomography (CT) scan revealed diffuse pleural effusion and pleural thickening in the left lung with no evidence of an intrapulmonary tumor (Figure 1(a)). Imaging findings were suggestive of malignant pleural mesothelioma. However, pleural effusion cytology was negative. Pleural thickening was confined to the left parietal pleura, allowing surgery for malignant pleural mesothelioma. Therefore, we performed endoscopic ultrasound with bronchoscope‐guided fine‐needle aspiration (EUS‐B‐FNA) on the left thickened pleura through the esophagus to minimize chest wall invasion (Figure 1(b)). The thickened pleura was pathologically diagnosed as lung adenocarcinoma as the tumor cells expressed TTF‐1, although they did not express calretinin and WT‐1 (Figure 1(c)). The patient was diagnosed with pseudomesotheliomatous carcinoma (PMC) of lung adenocarcinoma (cTXN0M1a stage IVA) based on the tumor progression. No driver gene mutations were found in the tumor sample, and chemotherapy was initiated.

**FIGURE 1:**
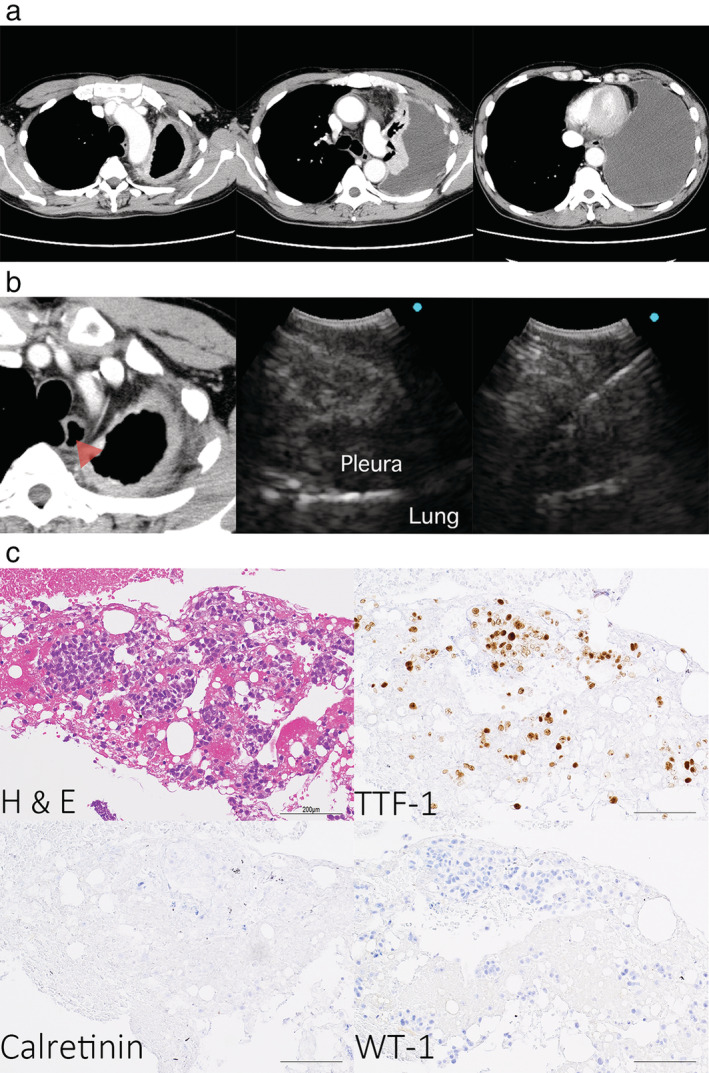
(a) Computed tomography (CT) findings. Enhanced chest CT revealed a large pleural effusion and pleural thickening in the left lung. (b) Endoscopic ultrasound with bronchoscope‐guided fine‐needle aspiration (EUS‐B‐FNA) findings. EUS‐B‐FNA was performed on the left thickened pleura. The image shows CT (left), and endoscopic ultrasound with bronchoscopy views of the left thickened pleura before (middle) and after (right) needle puncture. The red arrow indicates the ultrasound area. (c) Pathological examination of specimens. Hematoxylin and eosin staining and immunohistochemical analysis for TTF‐1, calretinin, and WT‐1. Tumor cells expressed TTF‐1 but not calretinin and WT‐1. Scale bar represents 200 μm

PMC is characterized by pleural effusion, pleural thickness, and histologically confirmed peripheral lung cancer infiltrating the pleura in a mesothelioma‐like manner.^1^ The rates of dissemination of malignant pleural mesothelioma were 4% and 22% using percutaneous lung biopsy and surgical biopsy, respectively.^2^ In pancreatic cancer, EUS‐FNA has a 2.2% peritoneal dissemination rate.^3^ Therefore, EUS‐B‐FNA may also reduce the risk of dissemination for PMC. PMC, diagnosed via thoracoscopy under local anesthesia, has also been previously reported. However, to the best of our knowledge, there have been no reports on PMC which have been diagnosed via EUS‐B‐FNA. In conclusion, EUS‐B‐FNA may be useful for the diagnosis of PMC because it is minimally invasive and a sufficient amount of tissue can be obtained for diagnosis.

## CONFLICT OF INTEREST

The authors declare that they have no conflicts of interest.
